# Molecular Basis of Mink ACE2 Binding to SARS-CoV-2 and Its Mink-Derived Variants

**DOI:** 10.1128/jvi.00814-22

**Published:** 2022-08-24

**Authors:** Chao Su, Juanhua He, Pengcheng Han, Bin Bai, Dedong Li, Jian Cao, Mingxiong Tian, Yu Hu, Anqi Zheng, Sheng Niu, Qian Chen, Xiaoyu Rong, Yanfang Zhang, Weiwei Li, Jianxun Qi, Xin Zhao, Mengsu Yang, Qihui Wang, George Fu Gao

**Affiliations:** a Department of Biomedical Sciences, City University of Hong Kong, Hong Kong, China; b CAS Key Laboratory of Pathogenic Microbiology and Immunology, Institute of Microbiology, Chinese Academy of Sciences, Beijing, China; c College of Life Sciences, Jiangxi Science and Technology Normal University, Nanchang, China; d College of Life Science and Technology, Southeast University, Nanjing, China; e University of the Chinese Academy of Sciences, Beijing, China; f College of Veterinary Medicine, China Agricultural University, Beijing, China; g School of Life Sciences, Shanxi University, Taiyuan, China; h School of Life Sciences, Division of Life Sciences and Medicine, University of Science and Technology of China, Hefei, Anhui, China; i College of Veterinary Medicine, Shanxi Agricultural University, Jinzhong, China; j Institutes of Physical Science and Information Technology, Anhui University, Hefei, China; k School of Laboratory Medicine and Life Science, Wenzhou Medical University, Wenzhou, China; l CAS Center for Influenza Research and Early-Warning, Chinese Academy of Sciences, Beijing, China; Peter Doherty Institute for Infection and Immunity

**Keywords:** SARS-CoV-2, mink, ACE2, Y453F, F486L, N501T, virus entry, cryo-EM structure

## Abstract

Severe acute respiratory syndrome coronavirus 2 (SARS-CoV-2) is transmitted between humans and minks, and some mutations in the spike (S) protein, especially in the receptor-binding domain (RBD), have been identified in mink-derived viruses. Here, we examined binding of the mink angiotensin-converting enzyme 2 (ACE2) receptor to mink-derived and important human-originating variants, and we demonstrated that most of the RBD variants increased the binding affinities to mink ACE2 (mkACE2). Cryo-electron microscopy structures of the mkACE2-RBD Y453F (with a Y-to-F change at position 453) and mkACE2-RBD F486L complexes helped identify the key residues that facilitate changes in mkACE2 binding affinity. Additionally, the data indicated that the Y453F and F486L mutations reduced the binding affinities to some human monoclonal antibodies, and human vaccinated sera efficiently prevented infection of human cells by pseudoviruses expressing Y453F, F486L, or N501T RBD. Our findings provide an important molecular mechanism for the rapid adaptation of SARS-CoV-2 in minks and highlight the potential influence of the main mink-originating variants for humans.

**IMPORTANCE** Severe acute respiratory syndrome coronavirus 2 (SARS-CoV-2) has a broad range of hosts. Mink-derived SARS-CoV-2 can transmit back to humans. There is an urgent need to understand the binding mechanism of mink-derived SARS-CoV-2 variants to mink receptor. In this study, we identified all mutations in the receptor-binding domain (RBD) of spike (S) protein from mink-derived SARS-CoV-2, and we demonstrated the enhanced binding affinity of mink angiotensin-converting enzyme 2 (ACE2) to most of the mink-derived RBD variants as well as important human-originating RBD variants. Cryo-electron microscopy structures revealed that the Y453F and F486L mutations enhanced the binding forces in the interaction interface. In addition, Y453F and F486L mutations reduced the binding affinities to some human monoclonal antibodies, and the SARS-CoV-2 pseudoviruses with Y453F, F486L, or N501T mutations were neutralized by human vaccinated sera. Therefore, our results provide valuable information for understanding the cross-species transmission mechanism of SARS-CoV-2.

## INTRODUCTION

Coronavirus disease 2019 (COVID-19) caused by severe acute respiratory syndrome coronavirus 2 (SARS-CoV-2) (1–4) has resulted in millions of deaths since December 2019 (https://covid19.who.int/) and continues to severely threaten human health (5). Previous studies indicated that SARS-CoV-2 has a broad range of hosts. In addition to humans, multiple mammalian species have been reported as susceptible to SARS-CoV-2 (6–13), including rhesus macaques, tigers, lions, cats, dogs, minks, ferrets, hamsters, and white-tailed deer.

The outbreak of SARS-CoV-2 on a farm with American mink (Neovison vison) was first reported in the Netherlands in April 2020 (14). In May 2020, Denmark, the largest mink pelt producer in Europe, reported infections on mink farms (15). Importantly, epidemiology studies suggested that the mink-originating SARS-CoV-2 could be transmitted between humans and minks (15, 16). Although a mink culling policy was implemented in the Netherlands and Denmark to prevent the transmission of SARS-CoV-2 on mink farms, SARS-CoV-2-infected minks were also found in other countries, such as Poland, the United States, and Latvia (17). The threat of SARS-CoV-2 infection in minks is ongoing.

The interaction of the spike (S) protein of SARS-CoV-2 and the angiotensin-converting enzyme 2 (ACE2) receptor protein determines viral entry into host cells and host tropism (18, 19). Mutations in the S protein, especially in the receptor-binding domain (RBD), can affect the adaptation of SARS-CoV-2 in the host by changing ACE2 binding or binding of neutralizing antibodies. For instance, the N-to-Y mutation at position 501 (N501Y) enables SARS-CoV-2 to bind to mouse ACE2 and supports the adaptation of SARS-CoV-2 to mice *in vitro* and *in vivo* (20, 21). The E484K mutation reduces antibody neutralization in humans (22). Some mutations in the RBD have been identified from mink- and ferret-originating SARS-CoV-2 (16, 23–25). The “cluster 5” variant, which carries the Y453F mutation in the RBD, was found in SARS-CoV-2 isolates from Danish farmed minks (23, 24). The Y453F mutation was reported to increase the entry efficiency of SARS-CoV-2 S into cells expressing mink ACE2 (mkACE2) or ferret ACE2 (24, 26) through enhanced binding to ACE2 (27) and enhance virus replication and morbidity in ferrets (24). Additionally, both the F486L and N501T mutations in the RBD have been identified in minks (16). Importantly, these mutations are also found in humans, and Y453F, F486L, and N501T can change the binding affinities to human ACE2 (hACE2) (27–30). It is important to understand the detailed binding mechanism of the mink-originating RBD mutations to mkACE2 and their potential influences on infectivity and immune escape in humans.

Here, we tested the binding of RBD with mink-originating and human-originating mutations to mkACE2. Furthermore, the cryo-electron microscopy (cryo-EM) structures of mkACE2 with RBD-Y453F or RBD-F486L provided a molecular basis for the Y453F and F486L mutations enhancing the binding forces in the interaction interface. Human vaccinated sera were observed to maintain the neutralizing activities against pseudovirus particles containing Y453F, F486L, or N501T. Taken together, these data highlight the entry mechanism of SARS-CoV-2 to mink cells and the potential influence of mink-originating variants in humans.

## RESULTS

### mkACE2 binding characteristics of mink-originating SARS-CoV-2 RBDs.

The genome of SARS-CoV-2 isolated from minks has been continually sequenced since farmed minks were first found to be infected by SARS-CoV-2. We downloaded these SARS-CoV-2 S protein sequences from the GISAID database (gisaid.org) and aligned them with the Wuhan-1 reference sequence (3). Considering that the RBD of S is responsible for binding the ACE2 receptor (18) and is the main target of neutralizing antibodies (31), we analyzed mutations in RBD from these S sequences. We found 14 combinations of mutations composed of nine residue substitutions (Table 1). Interestingly, single mutations Y453F, F486L, and N501T in RBD were identified in multiple countries, and the dominant strains were different among countries (Table 1). Moreover, another six combination mutations contained at least one of these three mutations, including RBD V367F/Y453F, RBD G446V/Y453F, RBD L452M/F486L, RBD F486L/N501T, RBD F486L/A520S, and RBD F486I/N501T. This finding implies that Y453F, F486L, and N501T mutations may result from the convergent evolution of SARS-CoV-2 in minks.

**TABLE 1:** Mutations in the mink-originating SARS-CoV-2 RBD from the GISAID database

Mutation(s) in RBD	Country	Dominant strain	No.
Y453F	Denmark	Δ69-70/Y453F/D614G	408
	Netherlands	Y453F	38
	USA	Y453F/D614G	9
	Lithuania	M153T/Y453F/D614G	4
	Poland	Q183R/Y453F/K558N/D614G/C1236F	4
F486L	Netherlands	A262S/Q314K/F486L/D614G	165
	USA	Δ69-70/Δ141-143/F486L/D614G/A701V	10
	Canada	F486L/D614G	1
N501T	USA	G142D/N501T/D614G	60
	Netherlands	N501T/D614G	5
	Denmark	N501T/D614G	3
	Latvia	L18F/A222V/N501T/D614G	1
F486I	Latvia	L18F/A222V/F486I/D614G	19
V367F/Y453F	Netherlands	V367F/Y453F	1
G446V/Y453F	Denmark	Δ69-70/G446V/Y453F/D614G	1
L452M/F486L	Netherlands	L452M/F486L/D614G	37
F486L/A520S	Lithuania	S254F/F486L/A520S/D614G	2
F486L/N501T	USA	G142D/Δ144/F486L/N501T/D614G	54
F486I/N501T	Latvia	L18F/A222V/F486I/N501T/D614G	1
V367F	Netherlands	V367F/D614G	5
N439K	Denmark	Δ69-70/N439K/D614G/G1223S	2
S477N	France	S477N/D614G	1
A520S	Lithuania	S254F/A520S/D614G	1

**FIG 1:**
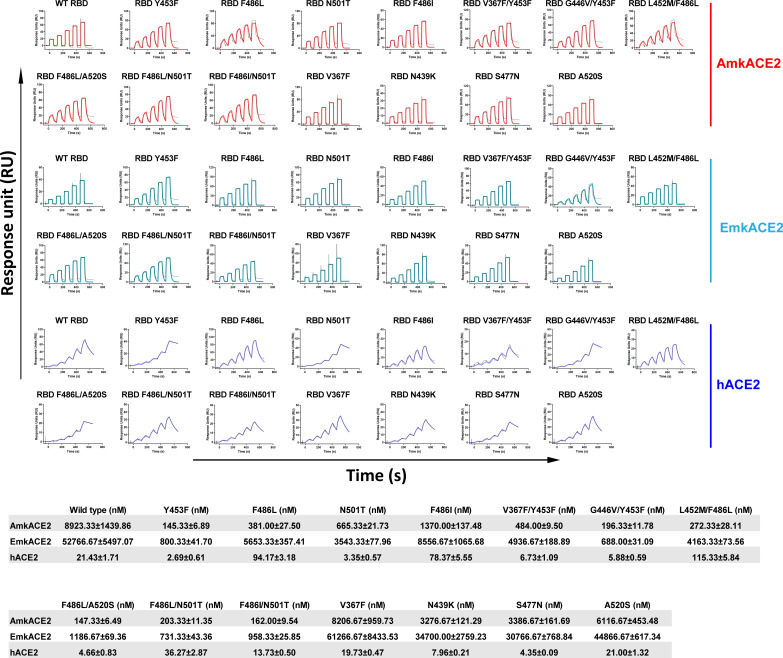
Binding affinities of mink-originating SARS-CoV-2 RBDs to ACE2s, characterized by surface plasmon resonance (SPR). Mouse Fc (mFc)-fused hACE2, AmkACE2, or EmkACE2 in the supernatant was captured in the CM5 chip by the preimmobilized anti-mFc antibody. Various concentrations of SARS-CoV-2 WT RBD, RBD Y453F, RBD F486L, RBD N501T, RBD F486I, RBD V367F/Y453F, RBD G446V/Y453F, RBD L452M/F486L, RBD F486L/A520S, RBD F486L/N501T, RBD F486I/N501T, RBD V367F, RBD N439K, RBD S477N, and RBD A520S protein were used to measure binding to AmkACE2, EmkACE2, or hACE2. Representative results from three experiments are shown. *K_D_* values are presented as means ± standard errors of the means (SEM) of three independent replicates (*n* = 3).

In order to explore whether these mutations in RBD affect the binding of RBD to mkACE2, we selected two types of mkACE2, American mink ACE2 (AmkACE2) and European mink ACE2 (EmkACE2), which belongs to the *Mustela* genus, with hACE2 was used as a control; we tested their binding affinities to RBD using surface plasmon resonance (SPR). There were seven amino acid differences between AmkACE2 and EmkACE2, located at positions 216, 309, 313, 354, 387, 660, and 667 (see Fig. S1A in the supplemental material). As shown in Fig. 1, wild-type (WT) RBD binds to AmkACE2 and EmkACE2 with equilibrium dissociation constants (*K_D_*) of ~8.92 μM and ~52.77 μM, respectively, representing a 6-fold difference between them. However, their binding affinities were much lower than that of hACE2 (~21.43 nM), with 416- and 2,462-fold differences, respectively. For the mutations, RBD with Y453F, F486L, or N501T exhibited a stronger binding affinity for both AmkACE2 and EmkACE2 than the WT RBD, especially RBD Y453F, for which binding was enhanced by more than 60-fold. Like RBD F486L, RBD F486I also showed a stronger binding affinity to mkACE2s. As expected, mink-originating combinations of mutations that contained Y453F, F486L, or N501T, including RBD V367F/Y453F, RBD G446V/Y453F, RBD L452M/F486L, RBD F486L/N501T, RBD F486L/A520S, and RBD F486I/N501T, also displayed higher binding affinities to mkACE2s than WT RBD. However, other mutations, such as V367F, N439K, S477N, and A520S, showed binding affinities similar to mkACE2s with WT RBD. Consistently, fewer sequences with the last four mutations have been uploaded to the GISAID database, suggesting limited transmission of these variants. Taken together, most of the mutations in RBD that occurred in minks, especially Y453F, F486L, and N501T, increased the binding ability of RBD to both mkACE2 receptors.

Considering the transmission of mink-derived SARS-CoV-2 to humans (15, 16), we also measured the binding affinities of these mutations to hACE2. All RBD mutations could still efficiently interact with hACE2. However, only the mutations of the residue at position 486, e.g., F486L, F486I, and L452M/F486L, resulted in reduced binding affinities to hACE2 compared to the WT RBD affinity. This is different from what was observed for binding to mkACE2s. Some other mutations, such as Y453F, N501T, V367F/Y453F, G446V/Y453F, F486L/A520S, N439K, and S477N, strengthened the interactions between RBD and hACE2 (Fig. 1). These findings suggested that the mink-derived variants displayed different binding characteristics to interact with mkACE2s versus hACE2, whereas they all still retained the ability to bind to hACE2.

### Binding characteristics of representative human-originating SARS-CoV-2 RBD variants to mkACE2s.

**FIG 2:**
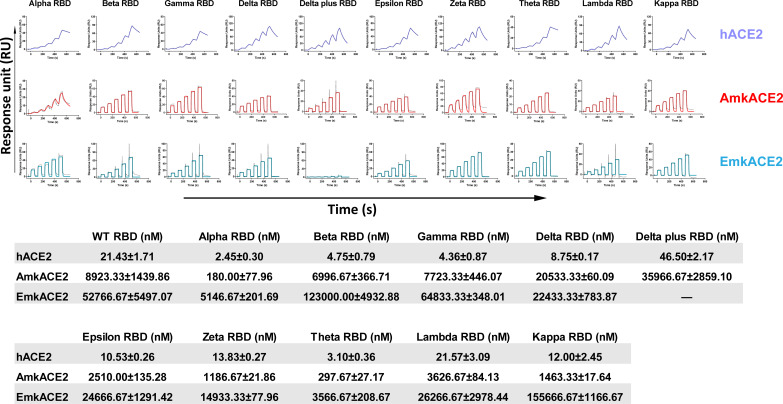
Binding of human-originating SARS-CoV-2 variants RBD to ACE2s. As shown in Fig. 1, SARS-CoV-2 Alpha RBD, Beta RBD, Gamma RBD, Delta RBD, Delta plus RBD, Epsilon RBD, Zeta RBD, Theta RBD, Lambda RBD, and Kappa RBD proteins were used to evaluate binding affinities to hACE2, AmkACE2, and EmkACE2. The representative results from three experiments are shown. *K_D_* values are means ± SEM of three independent replicates (*n* = 3). The *K_D_* values of WT RBD to ACE2s are from Fig. 1.

Many human SARS-CoV-2 variants have been identified since the start of the COVID-19 pandemic. Some have been deemed variants of concern by the World Health Organization (WHO) due to their potentially stronger transmission among humans (32). Next, we selected 10 representative human-originating variants, including the Alpha, Beta, Gamma, Delta, Delta plus, Epsilon, Zeta, Theta, Lambda, and Kappa variants (see Fig. S2), and explored whether these variants would still bind to mkACE2s. We prepared their RBD proteins and measured their binding affinities to hACE2 and mkACE2s. Consistent with a previous report (28), Alpha RBD, Beta RBD, Gamma RBD, and Theta RBD, which carry the N501Y mutation, displayed higher affinities for hACE2 (Fig. 2). Similarly, Alpha RBD and Theta RBD also had increased binding affinities to mkACE2s above that with WT RBD. However, Beta and Gamma RBDs, which carry an additional mutational residue position 417 compared with Theta RBD, had similar affinities with WT RBD to mkACE2s (Fig. 2). This finding suggests that the enhanced binding effect of the N501Y mutation to mkACE2s may be counteracted by the decreased effect caused by the K417N/T mutation. As expected, the Delta plus RBD that carries an additional K417N mutation than Delta RBD displayed a much lower binding affinity to mkACE2s than Delta RBD. In particular, it lost its ability to bind EmkACE2 (Fig. 2). In addition, Epsilon RBD, Zeta RBD, Lambda RBD, and Kappa RBD showed similar affinities for the hACE2 or mkACE2s compared with the WT RBD (Fig. 2). In other words, the important RBD variants have similar or increased binding abilities to mkACE2s, except for the Delta plus variant.

### The complex structure of AmkACE2 bound to SARS-CoV-2 RBD F486L or RBD Y453F.

**FIG 3:**
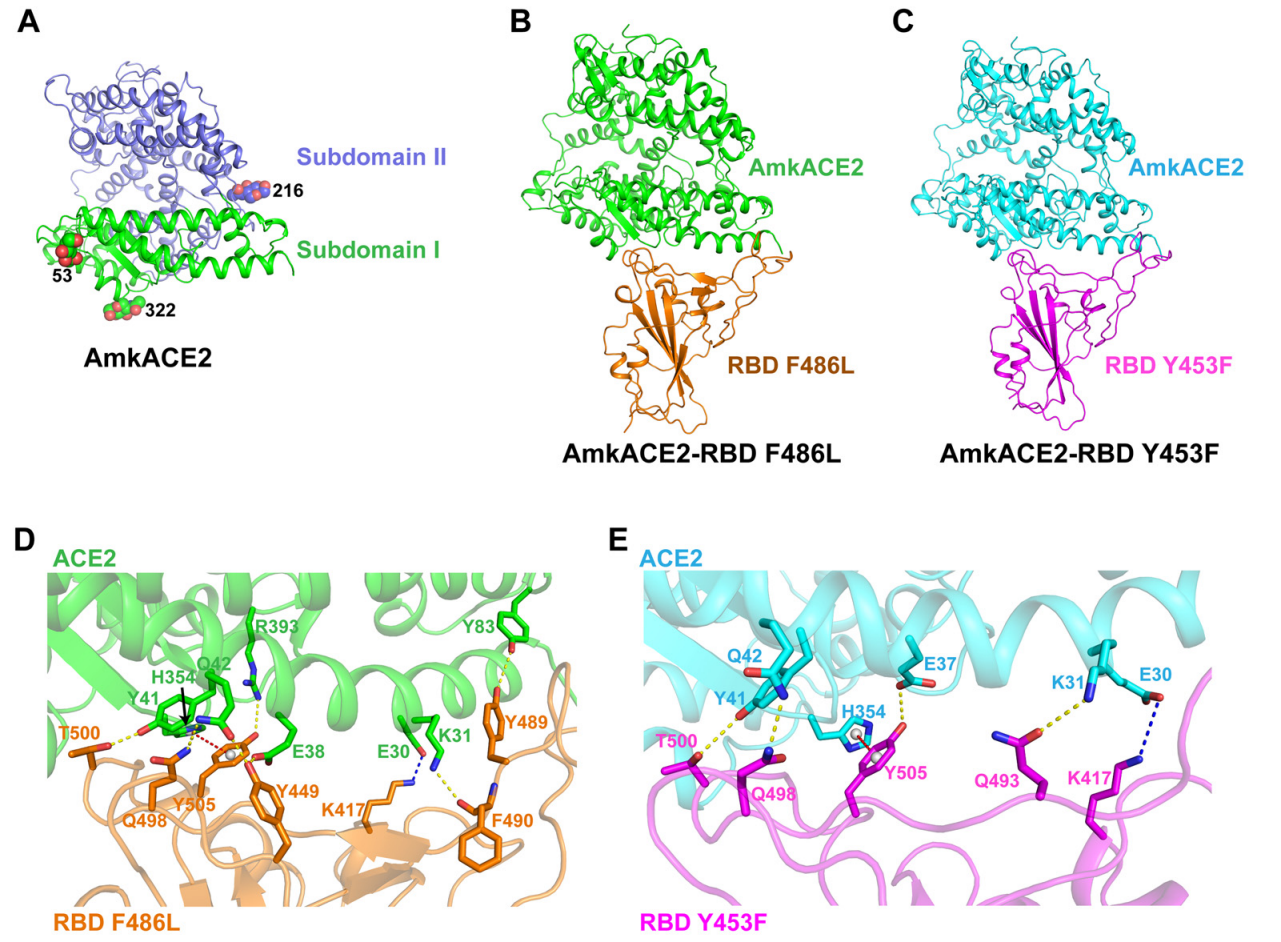
Complex structure of AmkACE2 bound to RBD F486L or RBD Y453F. (A) Cartoon structural representation of AmkACE2. Subdomain I (S19-Q102, N290-N397, and P415-E430) and subdomain II (S103-P289, E398-T414, and D431-D615) (57) are colored in green and blue, respectively. The glycan N-linked sites are labeled. (B and C) Cartoon structural representations of AmkACE2-RBD F486L (B) and AmkACE2-RBD Y453F (C). RBD F486L and RBD Y453F are colored in orange and magenta, respectively. (D and E) Detailed interactions between AmkACE2 and RBD F486L (D) or RBD Y453F (E). The key contact residues are shown as stick structures and labeled. Hydrogen bond interactions were analyzed at a cutoff of 3.5 Å and are colored in yellow. The salt bridge is colored in blue. The π-π stacking interaction is colored in red.

To further elucidate the mechanism of enhanced binding of mink-originating SARS-CoV-2 variants to the mkACE2s, we prepared the AmkACE2 peptidase domain (PD) and its complex with RBD, RBD F486L, or RBD Y453F proteins. However, due to the low binding affinity between AmkACE2 and RBD, we failed to obtain a stable complex of the two proteins. We finally obtained the 2.3-Å AmkACE2 crystal structure (Fig. 3A), 2.9-Å AmkACE2-RBD F486L complex cryo-EM structure (Fig. 3B; see also Fig. S3), and 2.85-Å AmkACE2-RBD Y453F complex cryo-EM structure (Fig. 3C; see also Fig. S3). The AmkACE2 structure consisted of two copies of the AmkACE2 molecule in one asymmetric unit with clear electron densities of residues S19 to D615 and three glycans N-linked to residues at positions 53, 216, and 322 (Fig. 3A). The overall structure of AmkACE2 PD contained two subdomains as found in other species’ ACE2: subdomain I and subdomain II (Fig. 3A), which is similar to hACE2, with a root mean square deviation (RMSD) of 0.459 Å (for 509 Cα atoms), in comparison to the hACE2 structure (PDB 1R42) (see Fig. S4A). Interestingly, a comparison between unbound AmkACE2 and its bound RBD F486L or RBD Y453F showed that the overall folds of AmkACE2 from AmkACE2 and AmkACE2-RBD F486L were very similar, with an RMSD of 0.520 Å (for 577 Cα atoms) (see Fig. S4B), while those from AmkACE2-RBD Y453F showed limited change, with an RMSD of 1.573 Å (for 594 Cα atoms) (see Fig. S4C). This finding indicated the conformational flexibility of AmkACE2 after binding to SARS-CoV-2 RBD.

We then dissected the binding details at the interfaces between AmkACE2 and RBD F486L or RBD Y453F. The residues located in the van der Waals contact distance (4.5 Å) between mkACE2 and RBD were selected, and the residues responsible for making hydrogen bond interactions were also identified (see Table S1). Although most of the residues in AmkACE2 that are involved in binding to RBD F486L and RBD Y453F are the same, the number of van der Waals interactions between AmkACE2 and RBD F486L is much greater than that between AmkACE2 and RBD Y453F (322 versus 157) (see Table S1), suggesting a moderate conformational change caused by the Y453F or F486L mutation in RBD. In addition, AmkACE2 formed 7 and 4 hydrogen bond interactions with RBD F486L and RBD Y453F, respectively (Fig. 3D and E). The hydrogen bonds between AmkACE2 and RBD F486L included AmkACE2 residue K31 interacting with RBD F490, E38 with Y449, Y41 with T500, Q42 with Y449 and Q498, Y83 with Y489, and R393 with Y505 (Fig. 3D). In contrast, two hydrogen bonds were the same in the AmkACE2-RBD Y453F structure, except for AmkACE2 E37 interacting with Y505 and K31 with Q493, including Y41 with RBD T500 and Q42 with Q498 (Fig. 3E). Moreover, AmkACE2 E30 was involved in forming the salt bridge with RBD K417 in both structures (Fig. 3D and E). This feature could explain why K417N/T mutations lead to lower binding affinities of SARS-CoV-2 RBD to AmkACE2 (Fig. 2). Furthermore, a π-π stacking interaction between AmkACE2 H354 and RBD Y505 also contributed to the AmkACE2-RBD interaction in both structures (Fig. 3D and E). Interestingly, sequence comparisons between AmkACE2 and EmkACE2 showed that only the residue at position 354 was different among the residues that are involved in van der Waals contacts with RBD (see Fig. S1A). EmkACE2 R354 could lose the π-π stacking interaction with RBD Y505, accounting for the observation that AmkACE2 showed a stronger binding ability to RBD than EmkACE2 (Fig. 1 and 2). Overall, the polar contacts mediated by hydrophilic residues contributed to the virus-receptor engagement.

### Comparison of binding interfaces between AmkACE2-RBD F486L and AmkACE2-RBD Y453F or hACE2-RBD F486L.

**FIG 4:**
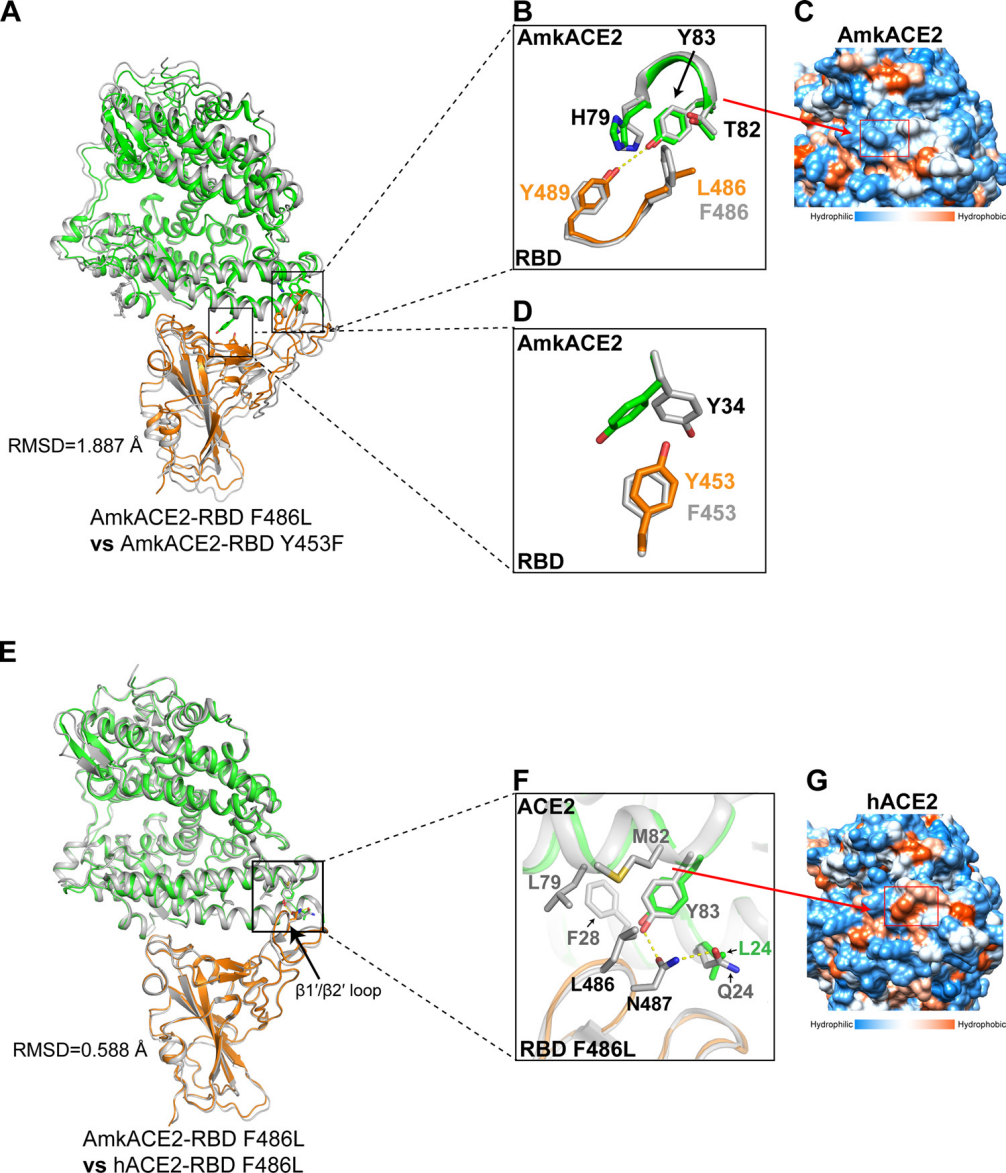
Structural comparison of AmkACE2-RBD F486L and AmkACE2-RBD Y453F or hACE2-RBD F486L. (A) Superimposition of AmkACE2-RBD F486L and AmkACE2-RBD Y453F. The RMSD is shown. AmkACE2 and RBD F486L in the AmkACE2-RBD F486L structure are colored green and orange, respectively. AmkACE2-RBD Y453F is colored gray. (B) AmkACE2 H79-Y83 and RBD F/L486-Y489 in both structures are superimposed. AmkACE2 H79, T82, and Y83, RBD F/L486, and Y489 are shown as sticks and labeled. Hydrogen bond interaction was analyzed at a cutoff of 3.5 Å and is colored in yellow. (C) The structure of AmkACE2 shows hydrophilic (blue) surfaces on the RBD binding face. A red rectangle indicates the surface of H79-Y83. Molecular surfaces are colored according to hydrophobicity, with blue, white, and orange corresponding to the most hydrophilic, neutral, and hydrophobic patches, respectively. (D) AmkACE2 Y34 and RBD Y/F453 in both structures are superimposed. AmkACE2 Y34 and RBD Y/F453 are shown as sticks and labeled. (E) Superimposition of AmkACE2-RBD F486L and hACE2-RBD F486L (PDB 7EKE). The RMSD and β1′/β2′ loop are shown. AmkACE2 and RBD F486L in the AmkACE2-RBD F486L structure are colored in green and orange, respectively. hACE2-RBD F486L is colored in gray. (F) ACE2 Q/L24, F28, L79, M82, and Y83, RBD L486 and N487 are shown as sticks and labeled. Hydrogen bond interaction was analyzed at a cutoff of 3.5 Å and colored in yellow. (G) The structure of hACE2 shows hydrophobic (orange) surfaces on the RBD-binding face. A red rectangle indicates the surface of F28, L79, and M82. Molecular surfaces are colored according to their hydrophobicity, with blue, white, and orange corresponding to the most hydrophilic, neutral, and hydrophobic regions, respectively.

To understand the effects of Y453F and F486L mutations on the AmkACE2-RBD interaction, the AmkACE2-RBD F486L structure was compared with the AmkACE2-RBD Y453F structure. Due to the limited overall conformational change, with an RMSD of 1.887 Å for 787 equivalent Cα atoms (Fig. 4A), we extracted the RBD residues near positions 486 and 453 and their binding residues in AmkACE2 for alignment. For the F486L mutation (Fig. 4B), AmkACE2 H79-T82-Y83 formed a hydrophilic groove (Fig. 4C), which clashed with the hydrophobic RBD F486 or L486 side chain. Compared with F486, the smaller L486 side chain showed less clash with AmkACE2, thus leading to a novel hydrogen bond between AmkACE2 Y83 and RBD Y489 in the AmkACE2-RBD F486L structure (Fig. 4B). Therefore, RBD F486L exhibited enhanced binding affinity to mkACE2s, and RBD F486I also had a similarly strong binding ability due to the similar I486 side chain with L486 (Fig. 1). In terms of the Y453F mutation (Fig. 4D), RBD F453 formed a stronger hydrophobic interaction with the benzene ring of AmkACE2 Y34 than RBD Y453, which contained a hydrophilic side chain (Fig. 4D). Thus, RBD Y453F displayed increased mkACE2 binding ability.

To elucidate the molecular mechanism underlying the lower binding affinity of RBD to AmkACE2 than hACE2, we further compared the AmkACE2-RBD F486L structure with the hACE2-RBD F486L structure previously reported by our group (PDB 7EKE). The majority of the secondary structure elements of the two structures could be well superimposed, with an RMSD of 0.588 Å for 732 equivalent Cα atoms (Fig. 4E). For the binding details, most of the AmkACE2 residues contributing to AmkACE2-RBD interactions were the same as those in hACE2, except for the residues that interacted with the RBD β1′/β2′ loop (Fig. 4E; see also Fig. S1A). hACE2 Q24 formed a hydrogen bond with RBD N487, while AmkACE2 L24 did not (Fig. 4F). Moreover, hACE2 F28-L79-M82 formed a hydrophobic groove (Fig. 4G), which was different from the hydrophilic groove in AmkACE2, and attracted the hydrophobic side chain of RBD L486 (Fig. 4F), leading to the formation of a hydrogen bond between hACE2 Y83 and RBD N487. Therefore, stronger polar contacts between hACE2 and RBD resulted in a higher binding affinity than AmkACE2-RBD.

### The transduction and immune-escape of mink-originating SARS-CoV-2 variants.

**FIG 5:**
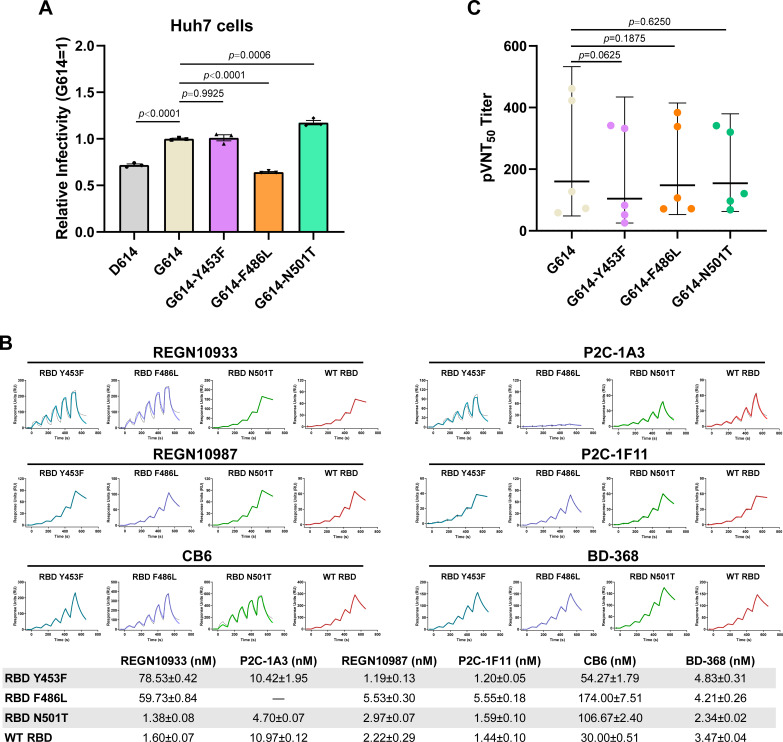
Entry and immune escape of SARS-CoV-2 variants. (A) The entries of SARS-CoV-2 D614, G614, G614-Y453F, G614-F486L, and G614-N501T pseudoviruses into huh7 cells were evidenced by cellular GFP expression. The representative results from three experiments are shown. Relative infectivity was normalized against that of the G614 pseudovirus. Statistical significance was analyzed using a one-way analysis of variance with Tukey’s multiple comparison test for multiple groups. (B) Antibody proteins in the supernatant were captured in the protein A chip. Serially diluted SARS-CoV-2 WT RBD, RBD Y453F, RBD F486L, and RBD N501T proteins were used to evaluate binding affinities to antibodies. *K_D_* values are means ± SEM of three independent replicates (*n* = 3). The representative results from three experiments are shown. (C) The pVNT_50_ against SARS-CoV-2 G614, G614-Y453F, G614-F486L, and G614-N501T pseudoviruses in sera from five volunteers who received three doses of ZF2001 vaccine. The horizontal bars show the geometric mean titer line with 95% confidential interval. Two-tailed Wilcoxon matched-pairs signed-rank test was used to analyze the neutralization values between different variants. The pVNT_50_ for each sample was determined twice.

According to reports elsewhere, the adaptive SARS-CoV-2 variants in minks, including those containing Y453F, can be transmitted to human (15, 16). Thus, we tested the influence of SARS-CoV-2 S Y453F, F486L, and N501T mutations on the entry into human cells. The same amount of pseudovirus carrying the SARS-CoV-2 variant S protein (including D614, G614, G614-Y453F, G614-F486L, and G614-N501T) were used to infect huh7 cells, and the expression of green fluorescent protein (GFP) by the pseudovirus was quantified for transduction efficiency. In agreement with a previous report (28), G614-N501T pseudovirus displayed a higher transduction efficiency than the G614 pseudovirus (Fig. 5A). Moreover, the SARS-CoV-2 Y453F pseudovirus exhibited similar transduction efficiency as the G614 pseudovirus, while F486L pseudovirus decreased the efficiency (Fig. 5A). This result suggested that the N501T mutation facilitates SARS-CoV-2 pseudovirus infections in humans, while the Y453F and F486L mutations do not.

Monoclonal antibodies (MAbs) are effective for treating COVD-19 patients. Many MAbs targeting SARS-CoV-2 have been used in human clinical trials (https://chineseantibody.org/opendata/dashboards/covid-19-mab-tracker). We expressed six antibodies that are in clinical trials and that target the receptor-binding motif of RBD, including REGN10933, REGN10987, CB6, P2C-1F11, P2C-1A3, and BD-368. Their binding strengths to RBD Y453F, RBD F486L, and RBD N501T were evaluated. As indicated in Fig. 5B, compared with WT RBD, RBD N501T maintained similar binding affinities to the six tested MAbs, with *K_D_* values that fluctuated within a 3-fold range, while F486L resulted in the complete loss of binding with P2C-1A3. This substitution also led to a decreased interaction with another two MAbs, REGN10933 and CB6. RBD F486L decreased the binding affinities to REGN10933 and CB6 by more than 35-fold and 5-fold, respectively. In terms of Y453F, it also affected the interaction with REGN10933, by nearly a 50-fold reduction of the binding affinities, compared with the WT RBD (Fig. 5B), which was in line with previous studies (33, 34). This result suggested that Y453F and F486L mutations may negatively influence the use of some MAbs in trials.

Next, we tested whether these three mutations affected the vaccine’s protective effect. The sera from five volunteers who received three doses of the ZF2001 vaccination were used to test the neutralization of G614-Y453F, G614-F486L, and G614-N501T pseudoviruses. As shown in Fig. 5C, no significant difference in 50% pseudovirus neutralization titers (pVNT_50_) were observed between G614 pseudovirus and variant pseudoviruses, indicating that the sera had similar neutralizing effects on G614-Y453F, G614-F486L, and G614-N501T pseudovirus with G614 pseudovirus. This suggests that vaccination is still an efficient method for protecting humans against these variants.

## DISCUSSION

The interaction between SARS-CoV-2 S and the ACE2 receptor is the first step in viral entry into host cells (18). The ability of SARS-CoV-2 S protein to bind to host ACE2s determines the host range for SARS-CoV-2 (35, 36). In this study, EmkACE2 showed a much lower binding affinity to SARS-CoV-2 than AmkACE2, due to the H354R mutation. This mutation exists widely in the ACE2 receptors of the ferret, stoat, North American river otter, sea otter, and hog badger (see Fig. S1B in the supplemental material). Ferret ACE2 and stoat ACE2, which have the same RBD-binding residues as EmkACE2, were reported as having no detected binding to the SARS-CoV-2 RBD (27), likely due to levels of binding between RBD and ACE2 that were too low to be detected by the BIAcore T200 machine (27). In support of this finding, ferrets have been reported to be infected with a large quantity of virus in lab settings (8, 37–40) and also in a high-exposure domestic setting (41). The American mink was proven susceptible to SARS-CoV-2 infection from humans and probably transmitted viruses back to humans, based on an epidemiology investigation (16). Furthermore, black-footed ferret ACE2 has the same RBD-binding residues as AmkACE2, indicating the potentially high risk of SARS-CoV-2 infection.

To adapt to the host, mutations in SARS-CoV-2 RBD that enhance binding to host ACE2 facilitate viral infection (20, 21). Compared with hACE2, the binding ability of mkACE2s to SARS-CoV-2 RBD is much lower. Thus, SARS-CoV-2 variants with higher binding affinities to the receptor would benefit viral entry and replication in minks and thereby acquire higher transmissibility. The majority of the mutations in RBDs observed in mink-origin SARS-CoV-2, including both single-point and double-point mutations, contribute to an enhanced binding affinity to mkACE2. The substitutions at position 486, including F486L, F486I, and the inclusive double-point mutations, strengthen the binding to mkACE2. In contrast, the exact same mutations in the RBD decrease the binding affinities to hACE2, suggesting a lower fitness in humans. This discrepancy suggests the adaptation of 486-containing variants in minks, providing the molecular evidence to support the spillback of related mink-origin SARS-CoV-2 variants to humans. Notably, the number of SARS-CoV-2 S sequences that contain F486L from the GISAID Initiative database decreased rapidly in humans after a cull policy was implemented in the Netherlands in October 2020 and in Denmark in November 2020 (see Fig. S5), which further indicates that F486L represents the adaption of SARS-CoV-2 to minks and subsequent spillback to humans.

Through structural analysis, we propose an underlying mechanism. Residue at position 486 interacts with the hydrophobic residues in hACE2 (L79-M82-Y83) but with hydrophilic residues in mkACE2 (T79-S82). Thus, F486, with a large hydrophobic side chain, favors the interaction with mkACE2 but decreases the interaction with hACE2. In addition, L486 or I486, due to a less hydrophobic side chain, thereby lowers the electronic clash with hACE2 and confers higher binding strength to the human receptor. Consistently, an RaTG13 RBD L486F mutant also displayed a decreased interaction with mouse ACE2, which contains the hydrophilic T79-S82 region (42).

In terms of the Y453F mutation, it confers increased binding not only to mkACE2 but also to hACE2, as previously reported (27–30). This is due to the stronger hydrophobic force of F453 (compared to Y453) with mkACE2 Y34, according to our structural data. H34 in hACE2 also forms a stronger hydrophobic interaction with RBD F453 than Y453 (28). Due to the broad presence of tyrosine or histidine at position 34 in the ACE2 receptors of domestic animals, such as dogs, cats, goats, and cattle (35), the Y453F mutation probably facilitates the spread of SARS-CoV-2 among these animals, which needs further study.

Based on the previously reported complex structures of different SARS-CoV strain RBDs bound with hACE2 or civet ACE2 (43, 44), the hydrophobic γ-methyl group of RBD T501 supports the mkACE2 K353-E38 or hACE2 K353-D38 salt bridges and thus stabilizes the binding interface, whereas the side chain of N501 does not. Therefore, the RBD N501T mutant increases the binding affinities with both mkACE2 and hACE2. In addition, the Alpha variant RBD enhances the binding to hACE2 by a π-π stacking interaction with hACE2 Y41 (28), which is conserved in mkACE2s. Nevertheless, RBDs of Beta, Gamma, and Delta strains maintain similar binding affinities to the WT strain to interact with mkACE2, indicating not better adaption in binding the mink receptor of these variants than the WT strain. Similar binding to mkACE2 may occur with the Omicron variant, which carries key mutations in the RBD similar to Beta variant, including N501Y, K417N, and E484A. Further studies are needed to evaluate these interactions.

American minks are widely farmed for fur in Europe, Asia, and North America (45), and so infected minks are a potential source of SARS-CoV-2 infection for humans. The N501T mutation benefits the infections of SARS-CoV-2 to human cells, which is consistent with the binding result, but the Y453F and F486L mutations do not. In this study, the data indicated that the neutralizing activities of ZF2001-vaccinated sera maintained a similar inhibition effect on pseudovirus harboring Y453F, F486L, or N501T mutation in the RBD region. However, the convalescent-phase sera exhibited slightly decreased neutralizing activities against Y453F-containing pseudovirus (27, 34), suggesting the superior protection efficacy that the vaccines stimulated compared to those induced by virus infection. Nonetheless, the similar neutralizing activities of vaccinated and convalescent-phase sera indicated the low possibility of breakthrough infection, in line with the epidemiology data that there was no more transmission of the Y453F, F486L, or N501 variants among humans after the culling policy was enacted.

In conclusion, characterization of the mkACE2-binding affinity of mink-originating and human-originating SARS-CoV-2 variants will help us understand SARS-CoV-2 transmission between minks and humans. The molecular features of SARS-CoV-2 RBD variants in binding to mkACE2 provide valuable information for understanding the cross-species transmission mechanism of SARS-CoV-2. In addition, it reminds us that vaccination effectively protects against mink-originating variants.

## MATERIALS AND METHODS

### Cells.

High Five cells (Invitrogen) and Sf9 cells (Invitrogen) were cultured in Insect-XPRESS medium (Lonza) at 27°C in a shaking incubator (120 to 130 rpm). Expi293F cells (Gibco) were cultured in SMM 293-TII expression medium at 37°C in a shaking incubator (140 rpm). HEK293T (ATCC), Vero (ATCC), and Huh7 (3111C0001CCC000679) cells were cultured at 37°C in Dulbecco’s modified Eagle medium supplemented with 10% fetal bovine serum.

### Gene cloning, expression, and protein purification.

The coding sequences of AmkACE2-mFc (residues 1 to 740; GenBank QPL12211), EmkACE2-mFc (residues 1 to 740; GenBank QNC68911.1), or hACE2-mFc (residues 1 to 740; GenBank NP_001358344) were inserted into a pCAGGS vector (MiaoLingPlasmid). The plasmids were transiently transfected into HEK293T cells by using polyethyleneimine (PEI), and after 48 h, the cell supernatants were concentrated for surface plasmon resonance (SPR) experiments.

The DNA sequences encoding the light chains and heavy chains of antibodies (including REGN10933 [PDB 6XDG], P2C-1A3 [PDB 7CDJ], REGN10987 [PDB 6XDG], P2C-1F11 [PDB 7E8M], CB6 [PDB 7C01], and BD-368 [PDB 7CHC]) were cloned into the pCAGGS vector. The light chain and heavy chain plasmids were together transiently transfected into HEK293T cells using PEI, and after 48 h, the cell supernatants were used for SPR experiments.

The DNA sequence encoding AmkACE2 (residues 19 to 615; GenBank QPL12211) with an N-terminal gp67 signal peptide sequence and a C-terminal 6-His tag sequence was cloned into the baculovirus transfection vector pFastBac1 (Invitrogen). The Bac-to-Bac baculovirus expression system was used to express the AmkACE2 protein. The pFastBac1-AmkACE2 plasmids were transformed into DH10Bac Escherichia coli cells to produce recombinant bacmids, and then the bacmids were transfected into Sf9 cells by using FuGENE 6 transfection reagent (Promega). Sf9 cells were used to amplify virus, and High Five cells were used to express the proteins. The supernatants were collected 72 h postinfection.

The DNA sequences encoding SARS-CoV-2 WT RBD (spike residues 319 to 541; GISAID EPI_ISL_402119) were inserted into the pCAGGS vector with an N-terminal interleukin-10 signal peptide sequence and a C-terminal 6-His tag sequence. The SARS-CoV-2 RBD variant plasmids (including RBD Y453F, RBD F486L, RBD N501T, RBD F486I, RBD V367F/Y453F, RBD G446V/Y453F, RBD L452M/F486L, RBD F486L/A520S, RBD F486L/N501T, RBD F486I/N501T, RBD V367F, RBD N439K, RBD S477N, RBD A520S, Alpha RBD, Beta RBD, Gamma RBD, Delta RBD, Delta plus RBD, Zeta RBD, Kappa RBD, Lambda RBD, Theta RBD, and Epsilon RBD) were constructed via site-directed mutagenesis using the Mut Express II Fast mutagenesis kit v2 (Vazyme). Recombinant RBD plasmids were transfected into Expi293F cells for protein expression using Sinofection transfection reagent (Sino Biological). Supernatants were collected 5 days posttransfection.

The supernatants containing AmkACE2 or RBD proteins were passed through a HisTrap HP 5-mL column (Cytiva). The target proteins were eluted in an elution buffer containing 20 mM Tris (pH 8.0), 150 mM NaCl, and 300 mM imidazole. AmkACE2 protein was then applied to a Resource Q anion-exchange chromatography equilibrated with buffer consisting of 20 mM Tris (pH 8.0), 50 mM NaCl, and eluted with an increasing linear gradient of 50 mM to 300 mM NaCl in 20 mM Tris (pH 8.0). AmkACE2 and RBD proteins were further purified by gel filtration chromatography on a HiLoad 16/600 Superdex 200PG column (Cytiva) in a buffer containing 20 mM Tris (pH 8.0), 150 mM NaCl. Purified AmkACE2 and RBD F486L (or RBD Y453F) proteins were mixed and incubated on ice overnight. The mixture was then purified on a HiLoad 16/600 Superdex 200PG column in 20 mM Tris (pH 8.0), 150 mM NaCl buffer.

### Cryo-EM sample preparation and data collection.

For cryo-EM, 4 μL purified AmkACE2-RBD F486L (or AmkACE2-RBD Y453F) complex protein at a 1.8-mg/mL concentration was applied to glow-discharged holey carbon grids (Quantifoil Au 1.2/1.3, 300 mesh). The grids were blotted for 3 s with a humidity of 100% and flash-frozen in liquid ethane using an FEI Vitrobot Mark IV. The data of cryo-specimens were collected using a Krios electron microscope operated at 300 kV. The microscope was equipped with a Falcon 4 using AutoEMation. Images were recorded in counting mode with 105,000× (96,000× for AmkACE2-RBD Y453F) nominal magnification, resulting in a physical pixel size of 0.669 Å/pixel (0.86 Å/pixel for AmkACE2-RBD Y453F). Each image was exposed for 2.53 s (6.51 s for AmkACE2- RBD Y453F) to lead to a total accumulated dose of 50 e-/Å^2^ which was dose-fractionated into 32 subframes. The defocus range of data sets varied from −1.0 μm to −2.0 μm.

### Image processing and 3D reconstruction.

MotionCor2 was used to correct the image drift and anisotropic magnification (46), and CTFFIND4 was used to estimate the initial contrast transfer function (CTF) parameters (47). Bad images were discarded based on CTF parameters during the initial screening. Particles were subsequently picked using Cryosparc with a similar process (48). Approximately 894,852 (1,243,791 for AmkACE2-RBD Y453F) particles were manually picked and applied to two-dimensional (2D) classification to generate templates for further particle autopicking. After 2D classification, the autopicked particles were used to generate the initial model in Cryosparc. After several rounds of 3D classification, a clean subset of 894,852 (410,404 for AmkACE2-RBD Y453F) particles were selected for the final 3D autorefinement. More details related to data processing are summarized in Table S2 in the supplemental material.

### Crystallization and data collection.

For crystallization, the AmkACE2 protein was concentrated to 15 mg/mL. Crystallization trials were carried out using a vapor-diffusion sitting-drop method with 0.8 μL protein mixed with 0.8 μL reservoir solution at 18°C. High-quality crystals were obtained in a solution containing 0.1M potassium thiocyanate and 30% (wt/vol) polyethylene glycol 2000 (PEG 2000)-MME2. Prior to collecting diffraction data, the crystals were flash-cooled in liquid nitrogen after briefly soaking in a reservoir solution supplemented with 20% (vol/vol) glycerol. X-ray diffraction data were collected at the Shanghai Synchrotron Radiation Facility (SSRF) BL02U1. The data sets were processed using XDS (49).

### Structure determination and refinement.

The AmkACE2 structure was determined via a molecular replacement method using Phaser (50) with the previously reported hACE2 structure (PDB 1R42) as a search model. The crystal structure of the SARS-CoV-2 RBD-hACE2 complex (PDB 6LZG) was fit into AmkACE2-RBD F486L and AmkACE2-RBD Y453F cryo-EM density maps using UCSF Chimera (51).The atomic model of each structure was built using WinCoot (52), and the refinement was performed using Phenix.refine (53). The stereochemical quality of the final model was assessed using MolProbity (54). Data collection, processing, and refinement statistics are summarized in Table S2 and Table S3. All structural figures were generated using PyMOL 2.3 software (https://pymol.org/2/).

### Surface plasmon resonance assay.

The SPR assays were performed to test the interactions between mFc-fused ACE2 (including hACE2, AmkACE2, and EmkACE2) and SARS-CoV-2 RBD or its variants (including RBD Y453F, RBD F486L, RBD N501T, RBD F486I, RBD V367F/Y453F, RBD G446V/Y453F, RBD L452M/F486L, RBD F486L/A520S, RBD F486L/N501T, RBD F486I/N501T, RBD V367F, RBD N439K, RBD S477N, RBD A520S, Alpha RBD, Beta RBD, Gamma RBD, Delta RBD, Delta plus RBD, Zeta RBD, Kappa RBD, Lambda RBD, Theta RBD, and Epsilon RBD) using a BIAcore 8K (GE Healthcare) with a CM5 chip (GE Healthcare) at 25°C in single-cycle mode. All proteins were in a buffer consisting of 10 mM Na_2_HPO_4_, 2 mM KH_2_PO_4_, 137 mM NaCl, 2.7 mM KCl, 0.005% Tween 20 (pH 7.4). The anti-mIgG antibody (Cytiva) was preimmobilized on the CM5 chip using standard amine coupling chemistry at a 50-μg/mL concentration. The soluble ACE2-mFc protein (hACE2, AmkACE2, or EmkACE2) in the supernatant was captured on the chip by the immobilized antibody. Serially diluted RBDs were used to evaluate ACE2 binding. After each reaction, the chip was regenerated with glycine (pH 1.7).

Similarly, the interaction between SARS-CoV-2 RBD variants (including RBD Y453F, RBD F486L, and RBD N501T) and monoclonal antibodies (including REGN10933, P2C-1A3, REGN10987, P2C-1F11, CB6, and BD-368) were also tested using a BIAcore 8K (GE Healthcare) with a protein A chip (GE Healthcare) at 25°C in single-cycle mode. The supernatant containing the antibody protein was captured on the chip, and serially diluted RBDs were used to evaluate antibody binding. After each reaction, the chip was regenerated using glycine (pH 1.5).

The equilibrium dissociation constants (*K_D_*) of each pair of RBD-ACE2 (or RBD-antibody) interactions were analyzed using a 1:1 binding model in the BIAcore Insight Evaluation 2.0.15.12933 software (GE Healthcare). All figures were generated using GraphPad Prism version 8.

### Production and quantification of pseudoviruses.

Pseudoviruses containing the backbone of a deficient vesicular stomatitis virus (VSV) vector (VSV-ΔG-GFP; BrainVTA) and SARS-CoV-2 variant S protein (including D614, G614, G614-Y453F, G614-F486L, G614-N501T) were generated using previously described protocols (28, 42). Briefly, 30 μg of the S plasmid with an 18-amino-acid deletion at the C terminus was transfected into 10-cm dishes containing HEK293T cells. Twenty-four hours later, the VSV-ΔG-GFP pseudoviruses were added to the cell supernatant and incubated for 2 h. The cell supernatant was then removed, and the cells were washed with phosphate-buffered saline (PBS) and cultured in media supplemented with anti-VSV-G antibody (produced by I1Hybridoma; ATCC CRL2700). Thirty-five hours postinfection, the supernatants were harvested, filtered (0.45-μm filter, Millipore catalog number SLHP033RB), aliquoted, and stored at −80°C.

The SARS-CoV-2 variant pseudovirus was first treated with 0.5 U/μL BaseMuncher endonuclease (Abcam) at 37°C for 1.5 h to remove the unpackaged RNA. Viral RNA was extracted using an RNA extraction kit (Bioer Technology). A quantitative reverse transcription-PCR assay was performed to quantify the viral RNA with a 7500 Fast real-time PCR system (Applied Biosystems). The primers and probe used to detect the L gene of the VSV virus were as follows: VSV-F, TGATACAGTACAATTATTTTGGGAC; VSV-R, GAGACTTTCTGTTACGGGATCTGG; VSV-probe, 6-carboxyfluorescein–ATGATGCATGATCCWGC–6-carboxytetramethylrhodamine.

### Pseudovirus infection assays and neutralization assays.

The SARS-CoV-2 variant pseudovirus was diluted to the same concentration as the VSV L gene for pseudovirus infection. Then, 100 μL of each pseudovirus was added to huh7 cells in 96-well plates. After 15 h, the number of GFP-positive cells was counted using a CQ1 confocal quantitative image cytometer (Yokogawa). Each group contained at least three replicates. Statistical analyses were performed using GraphPad Prism version 8.

For the pseudovirus neutralization assay, serially diluted sera were incubated with SARS-CoV-2 variant pseudovirus at 37°C for 1 h. The mixtures were then added to Vero cells in 96-well plates. After 15 h, the number of GFP-positive cells was counted using a CQ1 confocal quantitative image cytometer. Each group contained two replicates. Statistical analysis was performed using GraphPad Prism version 8.

### Statistical analysis of SARS-CoV-2 S sequence variants.

SARS-CoV-2 S protein sequences were downloaded from the GISAID Initiative database (gisaid.org) (55). Each sequence was aligned with the reference Wuhan-Hu-1 S sequence (GISAID EPI_ISL_402119) by using Mafft v7.310 (56). The S protein sequences isolated from mink and carrying mutations in the RBD (positions 319 to 541) were selected for further statistical analysis. In addition, the S protein sequences containing exact collection time and the Y453, F486L, or N501T mutation were also extracted for further analysis. The cumulative weekly number of variant sequences was calculated based on the collection time between 20 April 2020 and 25 November 2021.

### Data availability.

The cryo-EM density maps and corresponding atomic coordinates have been deposited in the Electron Microscopy Data Bank (EMDB) and Protein Data Bank (PDB), respectively. The accession numbers for the crystal and cryo-EM structures reported in this paper are as follows: AmkACE2 (PDB 7WA3), AmkACE2-RBD F486L (EMDB EMD-32379; PDB 7WA1), and AmkACE2-RBD Y453F (EMDB EMD-32358; PDB 7W8S). Source data are provided with this paper.
