# Supplementary material for: Molecular Basis of Mink ACE2 Binding to SARS-CoV-2 and Its Mink-Derived Variants
Source: J Virol. 2022 Aug 24;96(17):e00814-22. doi: 10.1128/jvi.00814-22 (PMC9472616; doi:10.1128/jvi.00814-22)
Supplement: Supplemental file 1 — Supplemental material. Download jvi.00814-22-s0001.pdf, PDF file, 1.2 MB [file jvi.00814-22-s0001.pdf]

Molecular basis of mink ACE2 binding to SARS-CoV-2 and its mink-derived variants

Chao Su[a,b], Juanhua He[b,c] Pengcheng Han[b,d], Bin Bai[b,e], Dedong Li[b,f], Jian Cao[b], Mingxiong Tian[b,g], Yu Hu[b,h], Anqi Zheng[b,d], Sheng Niu[b,i], Qian Chen[b,j], Xiaoyu Rong[b,k], Yanfang Zhang[b], Weiwei Li[b], Jianxun Qi[b], Xin Zhao[b,l], Mengsu Yang[a,#], Qihui Wang[b,#], George Fu Gao[a,b,c,#]

Supplemental Information

Table S1. Interaction between AmkACE2 and RBD F486L or RBD Y453F

AmkACE2	RBD F486L	RBD Y453F
S19	G476 (5), S477 (2)	
L24	A475 (4), G476 (2), N487 (7), Y489 (1)	A475 (3), G476 (2), N487 (5)
T27	F456 (9), Y473 (1), A475 (2), Y489 (6)	F456 (5), Y473 (1), Y489 (2)
F28	Y489 (7)	Y489 (6)
E30	K417 (9), L455 (4), F456 (8)	K417 (3), L455 (4), F456 (6)
K31	L455 (5), F456 (12), Y489 (8), F490 (3, 1), L492 (1), Q493 (1)	L455 (1), F456 (5), Y489 (5), F490 (1), L492 (1), Q493 (2, 1)
Y34	R403 (5), Y453 (20), Q493 (6), S494 (1), Y495 (5)	F453 (5), L455 (5), Q493 (5)
E35	Q493 (4)	
E37	Y505 (11)	Y505 (5, 1)
E38	Y449 (7, 1), S494 (1), Y495 (1), G496 (10)	G496 (5)
Y41	Q498 (9), T500 (7, 1), N501 (12)	Q498 (4), T500 (3, 1), N501 (3)
Q42	G446 (6), Y449 (6, 1), Q498 (6, 1)	G446 (1), Y449 (2), Q498 (1, 1)
L45	Q498 (3), T500 (2)	
H79	L486 (6)	F486 (7), Y489 (2)
T82	L486 (6)	F486 (2)
Y83	N487 (8), Y489 (1, 1)	N487 (1), Y489 (1)
N330	T500 (5)	T500 (2)
K353	G496 (3), N501 (12), G502 (3), Y505 (27)	G496 (2), Q498 (1), N501 (8), G502 (3), Y505 (18)
H354	D405 (1), G502 (7), Y505 (8)	G502 (6), Y505 (5)
D355	T500 (9), G502 (1)	T500 (5)
R357	T500 (3)	T500 (3)
R393	Y505 (4, 1)	
Total	322(7)	157(4)

The numbers in parentheses of RBD F486L or RBD Y453F residues represent the numbers of van der Waals contact the indicated residues conferred. The numbers with underline suggest numbers of potential hydrogen bonds between the pairs of residues. van der Waals contact was analyzed at a cutoff of 4.5 Å and hydrogen bonds at a cutoff

of 3.5 Å.

Table S2. Cryo-EM data collection and refinement statistics of AmkACE2-RBD F486L and AmkACE2-RBD Y453F

	AmkACE2-RBD F486L	AmkACE2-RBD Y453F
**Data collection**		
Magnification	105K	96K
Voltage (kV)	300	300
Electron exposure (e^-/ Å^2)	50	50
Micrographs collected (no.)	3546	3888
Defocus range (μm)	-1.0 ~ -2.0	-1.0 ~ -2.0
Pixel size (Å)	0.669	0.86
Symmetry imposed	C1	C1
Initial particle images (no.)	894,852	1,243,791
Final particle images (no.)	474,881	410,404
Map resolution (Å)	2.9	2.85
FSC threshold	0.35	1.5
**Refinement**		
Initial model used (PDB code)	6LZG	6LZG
Model composition		
Non-hydrogen atoms	6440	6455
Protein residues	790	788
Ligands	1	3
B factors		
Protein	50.79	38.54
Ligand	64.58	66.48
R.m.s. deviations		
Bond lengths (Å)	0.003	0.003
Bond angles (°)	0.575	0.591
**Validation**		
MolProbity score	1.32	1.40
Clashscore	2.39	0.87
Poor rotamers (%)	1.59	3.48
Ramachandran plot		
Favored (%)	97.20	96.56
Allowed (%)	2.80	3.44
Outliers (%)	0.00	0.00

Table S3. X-ray data collection and refinement statistics of AmkACE2

	AmkACE2
**Data collection**	
Space group	*P1211*
Cell dimensions	
a, b, c (Å)	67.697, 97.725, 105.063
α, β, γ (°)	90.0000, 91.9880, 90.0000
Resolution (Å)	71.54-2.28 (2.40-2.28)[a]
Unique reflections	61639
R_{merge} [b]	0.142 (0.591)[a]
R_{pim} [c]	0.064 (0.332)[a]
$I / \sigma I$	8.6 (2.4)[a]
$CC_{1/2}$	0.992 (0.737)[a]
Completeness (%)	98.2 (99.5)[a]
Redundancy	5.7 (3.9)[a]
**Refinement**	
Resolution (Å)	42.19-2.28
No. reflections	61059
R_{work} / R_{free} [c]	0.2275/0.2452
No. atoms	
Protein	9884
Ligand/ion	96
Water	326
B-factors	
Protein	39.76
Ligand/ion	76.63
Water	36.76
R.m.s. deviations	
Bond lengths (Å)	0.003
Bond angles (°)	0.568
Ramachandran plot	
Favored (%)	98.49
Allowed (%)	1.25
Outliers (%)	0.25

[a] Values in parentheses are for highest-resolution shell.

Supplementary Figure legends

A

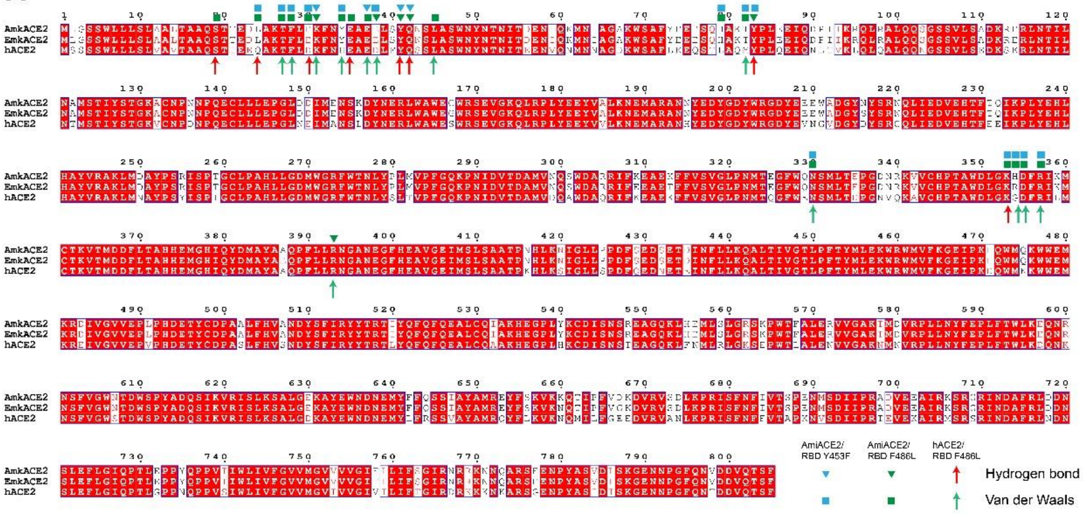

B

	19	24	27	28	30	31	34	35	37	38	41	42	45	82	83	330	353	354	355	357	393	Genus
American mink	S	L	T	F	E	K	Y	E	E	E	Y	Q	L	T	Y	N	K	H	D	R	R	*Neovison*
Black-footed ferret	S	L	T	F	E	K	Y	E	E	E	Y	Q	L	T	Y	N	K	H	D	R	R	*Mustela*
European mink	S	L	T	F	E	K	Y	E	E	E	Y	Q	L	T	Y	N	K	R	D	R	R	
Ferret	S	L	T	F	E	K	Y	E	E	E	Y	Q	L	T	Y	N	K	R	D	R	R	
Stoat	S	L	T	F	E	K	Y	E	E	E	Y	Q	L	T	Y	N	K	R	D	R	R	
North American river otter	S	L	T	F	E	K	Y	E	E	E	Y	Q	L	T	Y	N	K	R	D	R	R	*Lontra*
Sea otter	S	P	T	F	E	K	Y	E	E	E	Y	Q	L	T	Y	N	K	R	D	R	R	*Enhydra*
Hog badger	S	L	T	F	E	K	H	E	E	E	Y	Q	L	T	Y	N	K	R	D	R	R	*Arctonyx*
Chinese ferret badger	S	L	T	F	E	K	R	E	E	E	Y	Q	L	T	Y	N	K	H	D	R	R	*Melogale*

Fig. S1. Sequence analysis of ACE2 proteins. (A) Sequence alignment of ACE2 proteins from Amink, European mink (Emink), and human. The cyan and green square indicate amino acids in AmkACE2 that contribute to bind to RBD Y453F or RBD F486L *via* van der Waals, respectively. The cyan and green triangle indicate amino acids in AmkACE2 that are involved in interacting with RBD Y453F or RBD F486L through hydrogen bond, respectively. The red and green arrow indicate amino acids in hACE2 that are involved in interacting with RBD F486L *via* hydrogen bond and van der Waals, respectively. Sequence alignment was produced by ESPript [1]. The GenBank accession codes: AmkACE2: QPL12211; EmkACE2: QNC68911.1; hACE2: NP_001358344. (B) The nine species belonging to six genuses of Mustelidae are shown in the right column. Twenty-one residues of AmkACE2 which are crucial in interacting with the SARS-CoV-2 RBD are listed. Red letters indicate the substitutions in the ACE2

of eight species. The GenBank accession codes: Black-footed ferret: QNC68914.1; Ferret: NP_001297119.1; Stoat: XP_032187679.1; North American river otter: XP_032736029.1; Sea otter: XP_022374078.1; Hog badger: QLF98526.1; Chinese ferret badger: QLF98521.1.

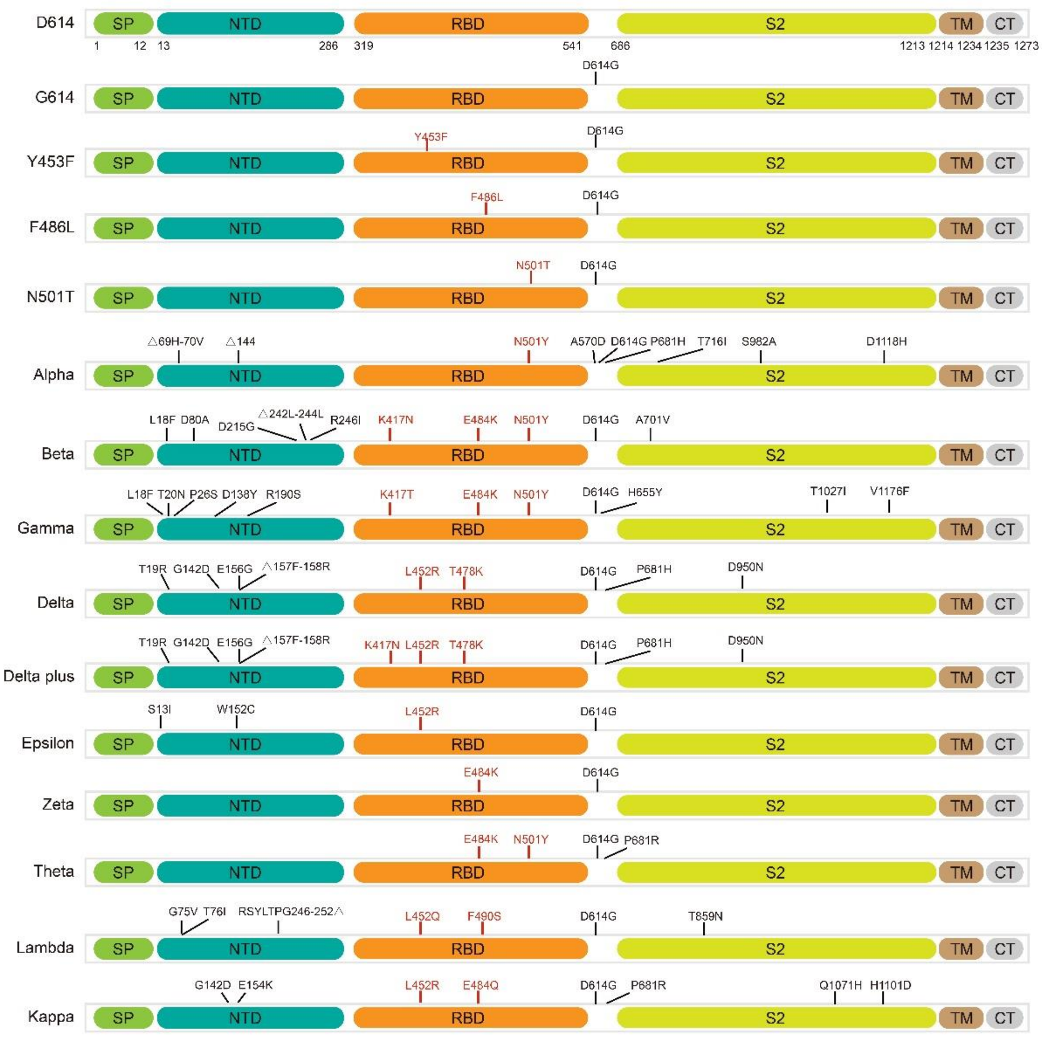

Fig. S2. Schematic representation of SARS-CoV-2 prototype (D614) and variant S proteins. The mutations of amino acids and positions are as indicated. SP: signal peptide, NTD: N-terminal domain, RBD: receptor-binding domain, TM: transmembrane, CT: C-terminal cytoplasmic tail domain.

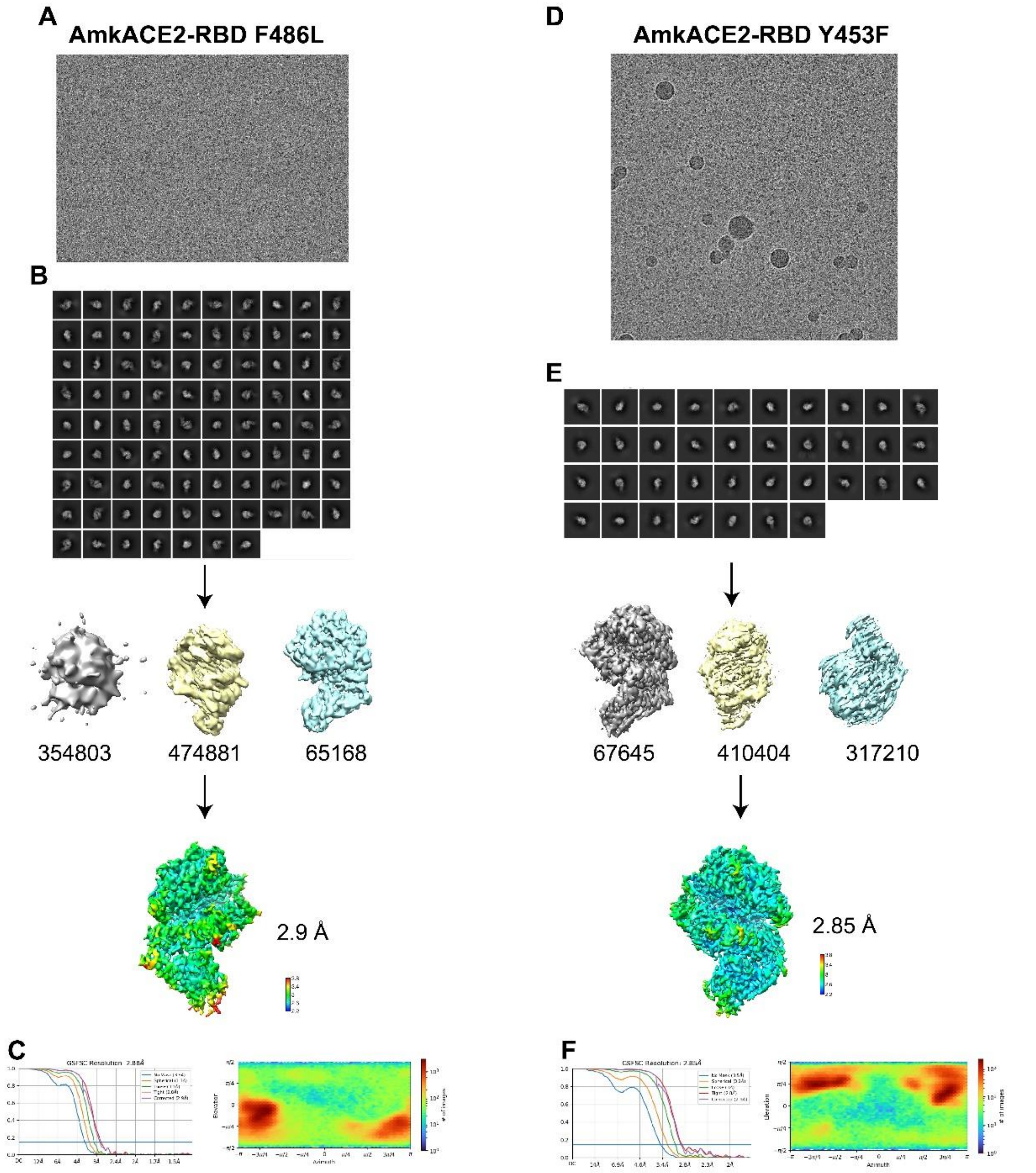

Fig. S3. EM data processing of AmkACE2-RBD F486L and AmkACE2-RBD Y453F complex. (A and D) One of raw cryo-EM micrographs. (B and E) Schematic to show steps in cryo-EM data processing. (C and F) FSC curve (left) and viewing direction distribution (right) of the map.

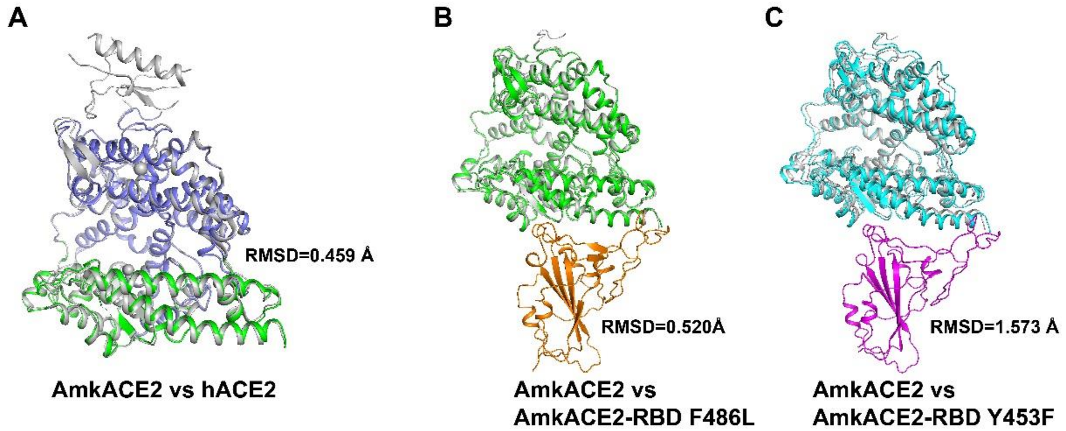

Fig. S4. Structural comparison of AmkACE2 with hACE2 or AmkACE2-RBD F486L or AmkACE2-RBD Y453F. (A) Superimposition of AmkACE2 and hACE2. RMSD is shown. hACE2 is colored in gray. Subdomain I and subdomain II of AmkACE2 are colored in green and blue, respectively. (B-C) Superimposition of AmkACE2 and AmkACE2-RBD F486L (B) or AmkACE2-RBD Y453F (C). AmkACE2 is colored in gray. RMSD is shown.

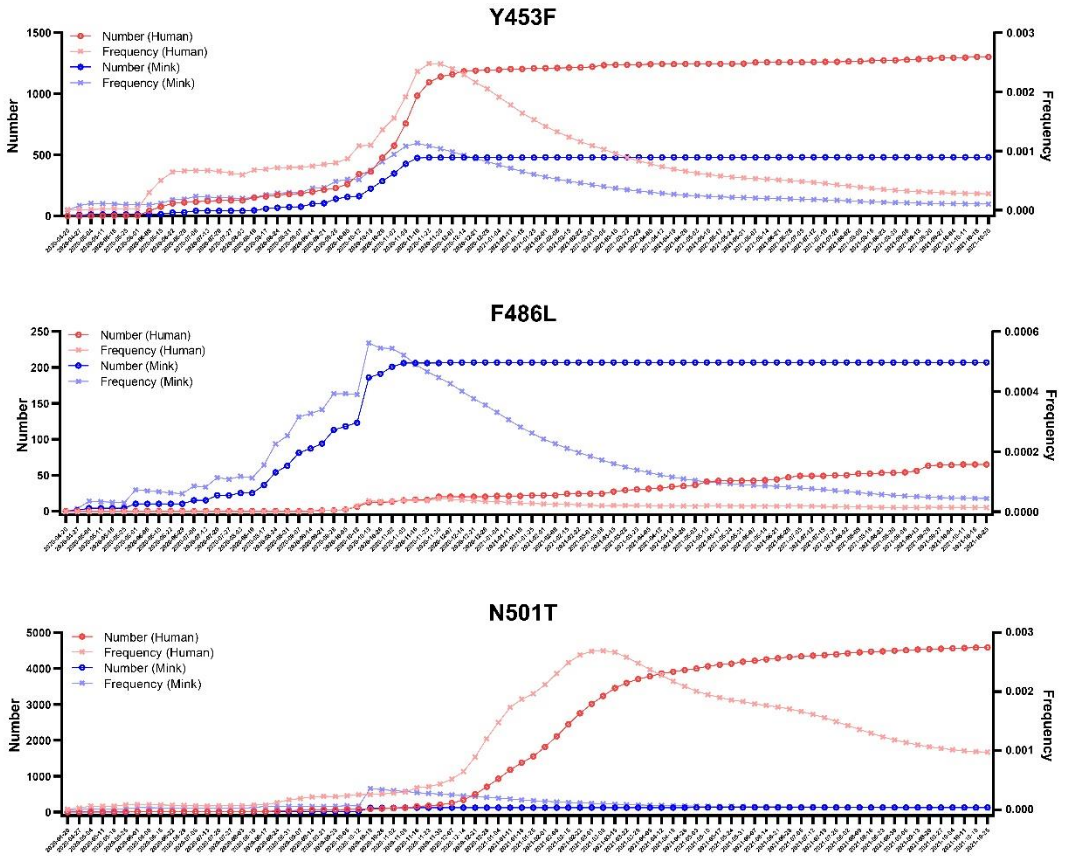

Fig. S5. Number and frequency of SARS-CoV-2 variant S sequences in the GISAID Initiative database. The cumulative weekly number and frequency of S sequence respectively carrying the Y453F (top), F486L (middle), or N501T (bottom) mutation in humans and minks were calculated between April 20, 2020 and October 25, 2021.

References

1. Robert, X. and P. Gouet, *Deciphering key features in protein structures with the new ENDscript server.* Nucleic Acids Res, 2014. **42**(Web Server issue): p. W320-4.
